# Specific entrustable professional activities for undergraduate medical internships: a method compatible with the academic curriculum

**DOI:** 10.1186/s12909-017-0980-6

**Published:** 2017-08-25

**Authors:** Alicia Hamui-Sutton, Ana María Monterrosas-Rojas, Armando Ortiz-Montalvo, Felipe Flores-Morones, Uri Torruco-García, Andrea Navarrete-Martínez, Araceli Arrioja-Guerrero

**Affiliations:** 1 Medical Education Secretary, Medicine School, Universidad Nacional Autónoma de México, Facultad de Medicina, Edificio B, 3er piso, Zip code, 04510 Coyoacán, Mexico; 2 Medical Internship Department, Clinical Teaching Secretary, Medicine School, Universidad Nacional Autónoma de México, Mexico City, Mexico

**Keywords:** Undergraduate internship, Entrustable professional activities, Benchmarks, Focus groups, Delphi technique, Rotations, Academic curriculum

## Abstract

**Background:**

Competency-based education has been considered the most important pedagogical trend in Medicine in the last two decades. In clinical contexts, competencies are implemented through Entrustable Professional Activities (EPAs) which are observable and measurable. The aim of this paper is to describe the methodology used in the design of educational tools to assess students´ competencies in clinical practice during their undergraduate internship (UI). In this paper, we present the construction of specific APROCs (Actividades Profesionales Confiables) in Surgery (S), Gynecology and Obstetrics (GO) and Family Medicine (FM) rotations with three levels of performance.

**Methods:**

The study considered a mixed method exploratory type design, a qualitative phase followed by a quantitative validation exercise. In the first stage data was obtained from three rotations (FM, GO and S) through focus groups about real and expected activities of medical interns. Triangulation with other sources was made to construct benchmarks. In the second stage, narrative descriptions with the three levels were validated by professors who teach the different subjects using the Delphi technique.

**Results:**

The results may be described both curricular and methodological wise. From the curricular point of view, APROCs were identified in three UI rotations within clinical contexts in Mexico City, benchmarks were developed by levels and validated by experts’ consensus. In regard to methodological issues, this research contributed to the development of a strategy, following six steps, to build APROCs using mixed methods.

**Conclusions:**

Developing benchmarks provides a regular and standardized language that helps to evaluate student’s performance and define educational strategies efficiently and accurately. The university academic program was aligned with APROCs in clinical contexts to assure the acquisition of competencies by students.

## Background

During the last decade, there has been an important development of the so called Entrustable Professional Activities (EPAs) to develop the medical competencies of students in clinical contexts. In his initial definition of EPAs Ten Cate describes them as “professional practice units defined as unsupervised tasks or responsibilities entrusted to students during their work after having acquired the competency level necessary to carry them out.” [1]. The intention is to connect the general doctor’s competencies (medical knowledge, clinical abilities and professional attitudes) with didactic activities and assessment during their clinical practices [2]. In the field of Health Sciences the Competency-based Education (CBE) movement has evolved internationally since the 90’s [3] from the mere enunciation of abstract principles to the detailed description of activities linked to compatible forms of assessment and feedback that complete the pedagogical circle of professional training.

The idea of EPAs has been welcome extensively in different medicine schools and departments throughout the world, and their theoretical, methodological, pedagogical and technical development has led to different educational experiences that have been documented in a variety of articles published in medical education literature. [1, 2, 4–10].

Since 2012, an interdisciplinary group of experts in medical education (MEDAPROC) at the Medicine School (MS) of the Universidad Nacional Autónoma de México (UNAM) has been working on operationalizing the CBE. In recent research [11] it was found that both teachers and students had difficulties to understand, implement and assess competencies in day to day educational activities.

Thus, the group went on to study a number of competency-based educational proposals such as the Bologna Pact [12], UNESCO [13] and OCDE [14] documents, as well as projects focused on medicine like the one made by the ACGME (Accreditation Council for Graduate Medical Education [15], the CanMeds (Canadian Medical Education Directives for Specialist, the Tuning project for Latin America [16] and others whose goal was to achieve learning results translated into competencies.

The following step was translating the abstract ideas of the competencies into concrete current academic programs. The group chose the Gynecology and Obstetrics 4th year course of the 2010 syllabus due both to its complexity (medical and surgical components) and the diversity of possible clinical scenarios [17]. They discussed the educational model (annex 1), and set its epistemological and pedagogical bases [18]. They also proposed a scheme to incorporate the courses’ content [17] and designed exercises to link practice with theory. The epistemological basis considered experiential theory and situational learning in the exercise of clinical reasoning that goes from inductive to deductive [19]: this supports active teaching strategies and the role of the educator as a guide for deliberate and reflexive practice from students [20].

In this model, the EPAs were translated as Actividades Profesionales Confiables (APROCs) term that, besides the translation, offers a particular way to operate the pedagogical model in clinical contexts in the Mexican National Health System.

In June 2014, when the AAMC (Association of American Medical Colleges) [21] published a document specifying 13 entrustable professional activities a graduate from medicine school should have in order to enter a residency program, the group decided to abide by this model to take advantage of it and align itself with the international CBE trends. Later that year the MEDAPROC group, after the experience of having designed and tested the model, allied with the medical internship area of the Clinical Teaching and Medical Internship Department (CTMID) of the UNAM MS to incorporate its pedagogical proposal into the undergraduate internship (UI).

The UI takes place during the tenth and eleventh semesters of the career, after having studied 2 years of theory at the MS facilities followed by two and a half years in clinical settings where they study several subjects. Interns go through six medical rotations spending 2 months in each: Surgery (S), Gynecology and Obstetrics (GO), Family Medicine (FM), Internal Medicine (IM), Pediatrics (P) and Emergency Medicine (ER). Unlike the previous semesters when learning was based on study, observation and performance of basic clinical procedures, during the UI the focus is on clinical practice, i.e., performing the acquired medical competencies under supervision. Thus, the main activities of their learning process happen within situational and reflexive learning.

The aim of this paper is to describe the methodology used in designing educational tools to assess competencies in the students’ clinical practice during their UI. Particularly the construction of specific APROCs in the S, GO and FM areas with three performance levels to be mastered by the end of the internship.

## Method

The study’s design used an exploratory mixed method [22], i.e., a qualitative first phase followed by a quantitative study. In the qualitative stage, information was obtained on both the expected and actual activities of interns in three rotations (FM, GO and S) through focus groups (FG). Benchmarks were developed from this data and its triangulation with other sources. These were later validated by professors using the Delphi technique. Once the APROCs were translated into reliable scales for each rotation, guidelines were created so that they could be used in the formative assessment of the interns’ clinical practice.

This study is part of the investigation protocol “A new model for medical education: education modules for the development of clinical competencies (MEDEC)” approved on April 2, 2013 and registered under number 033-2013 in the Ethics and Investigation Committee of the UNAM MS. Both professors and students were asked to give their informed consent in audiotape for the group interviews and in writing for the Delphi technique. Their identities were kept anonymous.

### Qualitative study

The qualitative study was divided into two steps; in the first one, FGs were completed to obtain the information about the activities that interns carry out in clinical contexts based on both theirs and their professors’ experience. In the second step the benchmarks were developed by triangulating various sources to compose performance level scales to evaluate the interns’ professional competencies in specific rotations.

#### Context

The field work was done by an interdisciplinary team of doctors, sociologists, students of the medical education master’s degree and social service interns in 2015 in the UNAM MS. That year, the Undergraduate Medical Internship Department (UMID) worked together with 52 clinics/hospitals from 5 different institutions both public and private around Mexico where 957 students were distributed into 48 groups in total.

From the 276 professors that collaborate with the UMID; those who participated in the FG were convened by the CTMID through an official written notice explaining the objective of such groups, i.e., to identify the contextual and pedagogical characteristics of the activities carried out by interns in their day to day clinical practice. In the case of students, social service interns who had completed their UI the year before and were assigned to the MS were convened.

Interview guidelines were developed to identify the compatibility between the academic (designed at the university) and operational program (designed at the clinic) with the activities an intern does in the FM, GO and S rotations. The guidelines were divided into three: 1) General questions regarding activities in each clinical space: What activities does an intern do in outpatient consultations? Which activities does he do on his own? Which require supervision? And from these, which must he be able to do on his own by the end of his outpatient services rotation? 2) Core topics of the academic curriculum. 3) Specific activities done by the intern during the rotation. This scheme was adjusted depending on whether it was aimed at teachers or students and according to the clinical areas each student went through in each rotation. The thematic contents also changed depending on each subject. Table 1 shows the interview guide for GO.

**Table 1:** Interview Guide

Service: Gynecology and Obstetrics	Area: Obstetric surgery Unit
1.- Which activities does the inter do in the surgery outpatient consult? 2.- Which activities does he perform on his/her own? 3.- Which activities require supervision? 4.- From these activities, which should the intern be able to perform on his own at the end of his outpatient consult rotation, considering they may be of great use during his social service?	
Main subjects	Activities
1. Labor 2. Physiologic and pathologic puerperium 3. Hemorrhaging during the second half of pregnancy 4. Pregnancy-induced hypertensive disorders 5. Pregnancy and diabetes 6. Premature rupture of membranes and pre-term labor 7. Breastfeeding and reproductive health.	• Clinical history (update or *di novo*) • Laborgram (Friedman curve, oxytocics, etc.) • Medical notes (admission, evolution, postpartum) • Informed Consent (analgesia, contraceptive method) • Physical exploration • Identification of risk factors • Information to relatives • Ultrasonic screening • Full labor care • Revision, manual or with instruments • Episiotomy, episiorrhaphy • Postpartum Exploration • Management of obstetric complications (uterine eversion, post-partum hemorrhaging, ruptured ectopic)

Both the voluntary informed consent and the interviews were recorded and transcribed. Based on Grounded Theory [23], a category tree was made to code and categorize transcriptions according to the activities performed by interns in specific rotations within clinical services.

Once transcriptions were coded, testimonies were ordered by category into two columns, one with the teacher’s comments and one with the student’s (annex 2). The analysis of the similarities and differences in the experiences, plus the diversity of stories in different locations and institutions allowed to create an accurate image of what was happening in the clinics and hospitals of the National Mexican Health System where the 10° and 11° semester UNAM MS students study.

#### Second phase qualitative study

The objective of the second stage was to build scales with narrative descriptions of the level of performance achieved by the interns in the typical activities of the UI in each rotation (benchmarks). The idea was to use the testimonies of the activities at the clinics gathered in the FG and write up improved narrative descriptions for the benchmarks used for assessment. As Hanson et al. explain, [4] to move from words to figures when evaluating allows to reconstruct the feedback process signaling both strengths and weaknesses.

When formulating the narrative descriptions, different sources of information were considered in order to formulate or inform the narrative construction [24] in such a way that they would reflect the interns’ level of performance. The considered documents were:

1. 2015 Internship Academic Programs (FM, GO and S) [25]
2. Operative Programs from some of the venues of the internship
3. ACGME *Milestone Project* from the FM, GO, and S residencies [15, 26, 27]
4. Document on the 13 EPA’s for the general physician from the AAMC titled “Core entrustable professional activities for entering residency AAMC: Developers Guide” [21]
5. Competencies of the 13 EPA’s outlined in the aforementioned document [21]
6. Testimonies by categories from the FG
7. Dreyfus and Dreyfus’s “Novice to expert” Model in the first three levels [28]
8. Table two from Ten Cate’s article “Nuts and Bolts of Entrustable Professional Activities” [1]
9. Miller’s Pyramid [29]
10. Bloom’s Taxonomy [30]

The procedure to develop the benchmarks started from the testimonies by category. Activities common to all rotations were eliminated to work only with the ones that were specific to each rotation. Once identified, they were both checked against the contents of each course’s 2015 academic program and revised considering the operative programs from some UI clinics. Keeping in mind the local scenario, we moved on to revise the AAMCs competencies per domain and their relationship to the APROCs.

While writing up the benchmarks for the now-called APROCs we considered three levels of performance according to Dreyfus and Dreyfus’s scale (novice, beginner and competent) and Miller’s Pyramid (know, know-how, and perform). Moreover, the recommendations in Ten Cate’s article describing EPAs were taken into consideration.

The next step in this iterative process to design the developmental benchmarks for each APROC was to use verbs from Bloom’s Taxonomy. These were chosen based on the expected performance for each level; for level 1 verbs from the knowledge and comprehension categories, for level 2 from the application category and for level 3 verbs from the analysis, synthesis and evaluation categories. There was flexibility in the use of verbs since some describe actions typical of medicine that are not found in Bloom’s taxonomy such as “diagnose,” “indicate”, “refer”, “prognosticate,” etc. [31].

The APROC’s titles refer to activities that translate into competencies and do not necessarily correspond to the topics in the programs. Once the APROCs were defined the narrative descriptors for each level were written using the present tense and avoiding verbs with negative connotation (lack, ignore, not know, overlook).

The APROCs for each rotation (FM, GO and S) were developed in subgroups and later revised and redesigned in plenary sessions until the final version was obtained. Twelve APROCs were created for GO, 14 for FM and 8 for S. They all reflect the expected learning outcomes in the specific internship rotations consistent with the graduate profile for the general physician as established in the 2010 Academic Curriculum. Table 2 shows an example of an APROC in each area.

**Table 2:** Examples of APROCs in each area

APROC 1 Surgery: To provide care to the patient with surgical wound		
Level 1	Level 2	Level 3
Skips asking the patient about risk factors relevant to the evolution of the surgical wound (background and comorbidities).	Identifies the patient’s risk factors and the evolution of the surgical wound through questions.	Asks directed questions to identify the risk factors for the evolution of the surgical wound.
APROC 3 Family Medicine: Prenatal and postnatal control		
Level 1	Level 2	Level 3
Acknowledges in an incomplete manner the importance of preconception medical attention and its components, participates passively in preconception medical care.	Provides partial preconception medical care to female patient and her partner, promotion of sexual health, healthy lifestyles and identifies reproductive risk factors. Initiates prophylactic supplements with folates, etc. Requires constant supervision.	Provides full preconception medical care to female patient and her partner, sex-ed, promotes healthy lifestyles and identifies reproductive risk factors. Initiates prophylactic supplements with folates, etc. In context, under reactive supervision.
APROC 2 Gynecology and Obstetrics: Labor care		
Level 1	Level 2	Level 3
Monitors and conducts labor using a laborgram.	Detects abnormalities during labor and proposes a confirmatory diagnostic and therapeutic plan. Asks for support from health team.	Handles an eutocic delivery from induction to episiorraphy, under supervision. Makes delivery, explores uterine cavity and makes episiorraphy. Identifies complications in next step.

### Quantitative study

To validate the final benchmarks for the three rotations involved the subgroups used the Delphi technique to reach the experts’ consensus, expressed as 80% or more of the desired level. Teachers from sites that had not participated in the FG received a personalized invitation to collaborate through the online surveys in the Limesurvey® platform. The goal was to reach agreements regarding the level of development, 1 = novice, 2 = beginner, 3 = competent, that interns had to achieve in the APROCs of each rotation. These three rounds of surveys with 13 GO specialists, 11 family doctors and 12 S professors took place in November and December 2015.

#### First round

Professors were contacted through email and phone calls in November and December 2015. A link was provided to access the survey and once in it there was an example showing how to answer it.

Based on the comments and observations of the first Delphi round some benchmarks were rewritten.

#### Second round

The comments of the first round were included in the second survey so that participants were aware of them. It was detected that professors were putting the actual level interns were achieving and not the level they were expected to achieve at the end of the rotation. Therefore, instructions were rephrased to read “Choose the level the intern must reach at the end of the 8-week rotation. Keep in mind levels are both progressive and inclusive; level 2 includes level 1; level 3 includes both levels 2 and 1.” At the end a space for general comments was added since some professors wrote down general observations in the space provided for specific comments about each activity. To identify each teacher’s answers (day and time) and to send personalized reminders to those who had not replied passwords were created to limit access to the survey.

#### Third round

The third round consisted of a personalized survey for each professor showing the levels chosen both by him and others in the previous rounds as well as the percentages and comments received. In the end, data was systematized and analyzed. Whenever one of the APROC’s levels reached at least 80%, consensus was given and hence, the APROC validated. Otherwise the APROC was discarded [32].

## Results

The results of the investigation can be described in two ways, curricular and methodological. From the curricular point of view, APROCs were identified in three UI rotations within clinical contexts in Mexico City and its surroundings; benchmarks were developed and validated by level. Table 3 shows the APROCs for FM, GO and S that resulted from this exercise.

**Table 3:** APP MEDAPROC in each rotation

APROC	Rotation		
	Surgery	Gynecology and Obstetrics	Family Medicine
1	Provide care to surgical wound	Give prenatal care to pregnant patient	Establish control of the patient with metabolic syndrome
2	Detect nontraumatic acute abdominal pain	Asses and assist in the care of the patient in labor	Administer vaccines
3	Attend patients with diabetic foot	Provide care and counseling to women during puerperium and breastfeeding	Provide prenatal and postnatal care
4	Identify vascular pathology of the lower limbs	Participate in the care of pregnant women with hemorrhage	Carry out control of healthy child under 5 years of age
5	Elaborate an early diagnosis of thyroid disease	Elaborate early diagnosis and initial care of patients with pregnancy hypertensive disorder.	Make patient’s family history/ background
6	Identify colon or anorectal disease	Elaborate early diagnosis and initial care of diabetic patients during pregnancy	Provide full care to patients with an infectious disease
7	Provide care to patients with urologic pathology	Participate in the care of pregnant women with premature rupture of membranes and premature labor threat	Provide full care to patients with an epidemiological surveillance disease
8	Participate in the surgical theatre	Provide care to patients with cervicovaginitis	Provide counseling on use of contraceptive methods
9		Provide care to women during menopause	Detect benign prostatic hyperplasia (BPH) and prostate cancer in patients at risk
10		Provide care to women with abnormal uterine hemorrhage	Detect uterine and cervical cancer (CaCu)
11		Detect uterine and cervical cancer	Detect neoplasm of mammary gland
12		Detect breast cancer in women	Carry out control of patients with musculo-skeletal disease
13			Detect behavior and mood disorders
14			Detect addictions

With the level-specific APROCs, guides were elaborated for both professors and students so that they could become familiar with the method and to define the results of the expected learning at the end of the rotation. Among other things, the guides explain how to assess the students’ performance during the course based on the narrative descriptions of each level. The “APP MEDAPROC” (Table 4) was designed to find and register the level reached by each student in his clinical practice; it is a useful tool for students, professors and education institutions for both the feedback and formative processes.

**Table 4:** “APP MEDAPROC” for Family Medicine

	Level 1	Level 2	Level 3
MF1	Overlooks to ask patient and relatives about risk factors. Hence, is unable to promote primary prevention measures. Forgets to take blood pressure and/or anthropometry (weight, height, waist circumference, etc.) correctly. Identifies isolated components of metabolic syndrome and automatically orders labs. Establishes a therapeutic plan at the request of other members of the health team, forgets the follow up plan. Makes physical exploration without identifying complications and indicates initial treatment following orders from others in the team. Refers patient to next care level at the health team’s command.	Identifies both in the patient and relatives some risk factors through the initial interview, but does not promote individual nor community primary prevention measures. Carries out both blood pressure measurement and anthropometry correctly, includes BMI and waist-hip ratio. Suspects metabolic syndrome diagnosis based on the presence of some of its components. Suggests labs and interprets them partially. Suggests therapeutic plan for some components and establishes follow-up plan without considering international standards. Suspects complications during physical examination and suggests initial treatment. Proposes referring patient without considering the current regulatory framework.	Identifies risk factors both in the patient and relatives through directed questions and promotes individual and community prevention measures. Takes blood pressure correctly and does complete anthropometry and interprets them. Diagnoses metabolic syndrome based on the presence of all four components (hypertension, diabetes mellitus, dyslipidemia and obesity). Requests and interprets labs for an accurate diagnosis. Establishes both a full therapeutic and follow-up plans in compliance with international standards for good control. Makes an early diagnosis and establishes initial treatment of complications. Refers patient opportunely under the current regulatory framework.
Self-assessment			
Highest- hierarchy resident			
Physician assigned to service			
Rotation coordinator			

From the methodological point of view this research fostered the development of a model to build APROCs from six mixed methods, summarized as follows:

1. Study of international literature about competency- based education and EPAs.
2. Analyze the MEDAPROC proposal and its compatibility with the specific curricular programs.
3. Make FG with professors and students to identify the real and the expected activities in the courses of interest.
4. Order and organize the information to obtain a document with testimonies for each category distinguishing between professors and students to identify the APROCs.
5. “Triangulate” the information obtained with other sources to make narrative descriptions for three levels (benchmarks) in each APROC; revise them individually and collectively to get a final version.
6. Validate the benchmark scales for the APROCs through the Delphi technique to achieve both experts’ consensus and reliability of the instruments for assessment.

## Discussion

The original objective, to describe the methodology used in the design of specific APROCs as educational tools to assess competencies in the clinical practice of interns based on the MEDAPROC, was achieved. Moreover, benchmarks were developed for three rotations of the UI. The results went beyond explaining EPAs with definitions and description of activities, the APROCs were made compatible with the academic and operational UI programs (CTMID, MS, UNAM) and were adapted to the clinical contexts of the Mexican National Healthcare System.

A similar effort to link CBE to clinical scenarios was made in the Netherlands [2] where a method was developed to achieve such relationship by developing pedagogical designs. The difference is that, in our study, the exercise was made from the university to be implemented in multiple clinics and hospitals; and, in other studies described in literature, the development of EPAs was made in specific clinical environments. The question that arises is whether the UI’s APROCs work the same in different sites or whether each clinic or hospital needs specific criteria to apply the APROCs.

There are published works that describe the steps followed for the construction of EPAs, the most influential being that of Ten Cate [1]. Aylward et al. [7] also contribute to this topic in their article explaining how they planned and implemented an EPA related to the shifts of Pediatric residents. In Aylward’s EPA, as in this study, three-level scales were developed to measure the reliability achieved in the competencies. Recently in Australia, Kwan et al. [5], have described five steps to build EPAs and presented the development of two EPAs for the Emergency Room service, also constructed with three-level scales. As in our study, they used FG, but they did not validate the benchmarks through the Delphi technique as was done in this and other cases [33–35].

As experiences in the construction of EPAs are registered, the concern to ensure their quality has aroused and validation strategies have been put forward to ensure the reliability of the scales. Chen et al. [10], propose a methodology that considers seven aspects that must be attended when developing an EPA. Even though many of them were implemented in the construction of our APROCs, we still need to test the benchmarks’ functionality as a supervision tool and as a resource for formative assessment of the UI. Post et al. [8] presented an instrument to review EPAs that takes into consideration seven aspects that were useful when testing the viability of EPAs in pilot studies.

When work on the benchmarks started [36] emphasis was made on the specialties of medical residencies; but it has been proven that [6] EPAs can also be implemented in the undergraduate level as in the case of the UI, with the advantage that it gives continuity to both the contents and the methodology proposed for the acquisition of medical competencies. Benchmarks provide a standardized language that helps eliminate personal and institutional interpretations about the student’s performance. The process allows professors to assess knowledge, skills and abilities in each benchmark as well as define the strategies to evaluate students efficiently and accurately [31]. The APROCs constitute an attempt to align the learning results established by the current core curriculum of the MS at UNAM with international trends.

Even though the UNAM MS is the largest medicine school in Latin America and has more than 50 clinics and hospitals in its UI, this study was carried out in only one educational organization, and the investigation should be expanded to other universities. Another limitation was time. The UI-specific APROCs, unlike those of the medical residency, must be developed in a 2-month periods. This hastens its teaching in clinical situations that do not always promote learning. Among the pending tasks, we must test the proposal to assess how professors and students adapt to the new pedagogical model to later adjust the scales, formats and processes for a more efficient implementation.

In regards to the Delphi technique we found that there is no single definition of the term “consensus” and that there is a variety of methods to implement it [37]. In this work, due to the small samples and the logistic complications of assigning numerical scales to the three levels, we did not use validity coefficients to measure the degree of consistency of the results obtained (Pearson, Kappa) nor did we use descriptive statistics measures (mean, median, variance, standard deviation, confidence intervals) in the result analysis of the Delphi technique; we merely presented the percentages of the answers that were obtained.

## Conclusions

To our knowledge, this study is the first formal effort to incorporate APROCs in an UI in Mexico as well as a methodology based on the CBE pedagogical model to develop useful benchmarks for the supervision of clinical practice and formative assessment. The APP MEDAPROC for the UI will enable the planning of the teaching-learning process through previous instructional design based on experiential theory, situational learning, deliberate practice and reflection. Making the APROCs operational in accordance with the 2010 Academic Curricula of the UNAM MS constitutes a powerful educational tool in clinical environments that contributes to the improvement of the quality of medical care and patient security. Nevertheless, the benchmarks should not be used as the only instrument to assess the acquisition of knowledge, skills and attitudes in the specific activities of the UI rotations; it is necessary to combine them with other assessment strategies that show the development of the interns’ professional competencies.

## Figures and Tables

**Annex 1:**
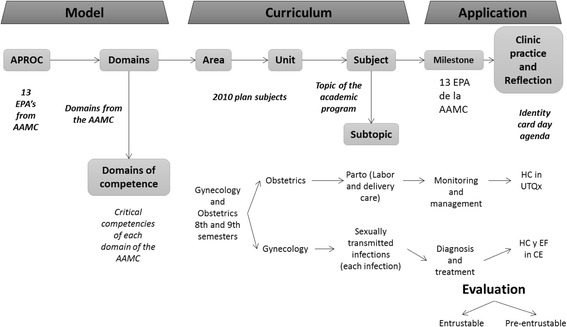
Model from MEDAPROC

## Appendix

**Annex 2:** Testimonies by category

Testimony		
Category	Students	Teachers
3.2.4 Attention of labor	I was one of the doctors who made more deliveries but they were only 3. I did not do it alone, I was always helped. HGV/IMSS/GO/28042015 You made the delivery, turned the baby to the pediatrics personnel and were supervised during the episiotomy; but really you were in charge.HGEBC/ISSSTTE/GO/280515 Did you make a delivery? Yes, we made all of them except during the first week. It was to learn. HGZ4 7/IMSS/GO/280415 In the deliveries that took place, the intern learns how to handle it; not the labor and everything it involves, for example administering oxytocin, draw blood from the patient, measure the baby’s heartbeat. We learn how to use and handle oxytocin, to channel patients, basically what labor is in the Obstetric Surgery Unit.HGDFQ/ISSSTE/GO/050515	This must always be supervised by their superiors, residents or attendings. I always tell them “you can examine the patients, you are free to do so, but ask always and if possible have an attending present”. That way we can compare what you say to what I see in the patient’s examination and of course learn to make a delivery adequately which is really important. Here you are working in a more practical way, as the saying goes, practice makes a surgeon. Practice makes you learn things better. You can revise one and ten times the theory, but in practice if you do things right and learn how to do them right then you learn better. So, it is important that the students are supervised either b attendings or residents and thus learn in the best possible way HGZ47/IMSSGYO//130,515 Since there are no residents they make the deliveries. At the beginning, they might feel anxious because they have very little experience, but in the end the truth is that the students make the whole procedure by themselves during their shifts. HGMORELOS/ISSSTE/GYO/130515

## Abbreviation

CBE: Competency-based education
CTMID: Clinical Teaching and Medical Internship Department
EPAs: Entrustable Professional Activities (APROCs for the Mexican version)
ER: Emergency Medicine
FG: Focus Groups
FM: Family Medicine
GO: Gynecology and Obstetrics
IM: Internal Medicine
MS: Medicine School
P: Pediatrics
S: Surgery
UI: Undergraduate Internship
UMID: Undergraduate Medical Internship Department
UNAM: Universidad Nacional Autónoma de México
